# Human Schwann cells are susceptible to infection with Zika and yellow fever viruses, but not dengue virus

**DOI:** 10.1038/s41598-019-46389-0

**Published:** 2019-07-09

**Authors:** Gaurav Dhiman, Rachy Abraham, Diane E. Griffin

**Affiliations:** 0000 0001 2171 9311 grid.21107.35 W. Harry Feinstone Department of Molecular Microbiology and Immunology, Johns Hopkins Bloomberg School of Public Health, 615 North Wolfe Street, Baltimore, MD 21205 USA

**Keywords:** Pathogens, Dengue virus, Viral pathogenesis, Peripheral nervous system

## Abstract

Zika virus (ZIKV) is a re-emerged flavivirus transmitted by *Aedes spp* mosquitoes that has caused outbreaks of fever and rash on islands in the Pacific and in the Americas. These outbreaks have been associated with neurologic complications that include congenital abnormalities and Guillain-Barré syndrome (GBS). The pathogenesis of ZIKV-associated GBS, a potentially life-threatening peripheral nerve disease, remains unclear. Because Schwann cells (SCs) play a central role in peripheral nerve function and can be the target for damage in GBS, we characterized the interactions of ZIKV isolates from Africa, Asia and Brazil with human SCs in comparison with the related mosquito-transmitted flaviviruses yellow fever virus 17D (YFV) and dengue virus type 2 (DENV2). SCs supported sustained replication of ZIKV and YFV, but not DENV. ZIKV infection induced increased SC expression of IL-6, interferon (IFN)β1, IFN-λ, IFIT-1, TNFα and IL-23A mRNAs as well as IFN-λ receptors and negative regulators of IFN signaling. SCs expressed baseline mRNAs for multiple potential flavivirus receptors and levels did not change after ZIKV infection. SCs did not express detectable levels of cell surface Fcγ receptors. This study demonstrates the susceptibility and biological responses of SCs to ZIKV infection of potential importance for the pathogenesis of ZIKV-associated GBS.

## Introduction

Zika virus (ZIKV) is a plus-strand enveloped RNA virus that belongs to the genus *Flavivirus* and is related to other mosquito-borne flaviviruses, including dengue, yellow fever, West Nile, Japanese encephalitis and Spondweni viruses^1^. *Aedes* spp. mosquitoes are the main vectors for ZIKV, yellow fever virus (YFV) and all four types of dengue virus (DENV) but ZIKV can also be transmitted through infected semen and from mother to fetus^2–4^. ZIKV was first isolated from primates in Uganda in 1947, from *Aedes africanus* mosquitoes in 1948^5^ and from a febrile man working in Uganda in 1963^6^. For the next five decades sporadic cases of benign, self-limiting acute illness characterized by fever, headache, myalgia and rash were identified in Asia and Africa^7–9^.

The first recognized large outbreak of human ZIKV infection occurred in 2007 on the Pacific island of Yap, in the Federated States of Micronesia^10^. Subsequent outbreaks beginning in October 2013 and continuing throughout 2014 were recognized across Oceania in four groups of Pacific islands: French Polynesia, Easter Island, the Cook Islands, and New Caledonia with 8,750 reported cases^11^. In May 2015, locally acquired ZIKV disease was recognized in Brazil^12^ followed by rapid spread through the Americas and Caribbean islands with accompanying reports of neurological complications and congenital malformations including microcephaly^13–15^.

The first association of ZIKV infection with Guillain-Barré syndrome (GBS) was a retrospective study of the French Polynesian outbreak where there was a 20-fold increase in GBS incidence and all of the 42 patients hospitalized with GBS had anti-ZIKV antibody^16^. During the ZIKV outbreak in South America, 66 of the 68 patients with GBS in Colombia had symptoms compatible with ZIKV infection before the onset of GBS^17^ and in Martinique, a prospective study determined that the incidence rate of GBS during the 2016 ZIKV outbreak was 4.52 times that of prior years^18^. In 2016, the World Health Organization officially recognized ZIKV as a cause of GBS and microcephaly^19^.

GBS, a potentially life-threatening peripheral nerve disease, is characterized by a rapid onset of bilateral weakness progressing to paralysis that may be accompanied by sensory symptoms. GBS typically occurs 1–3 weeks after an infectious disease postulated to trigger a pathogen-specific immune response that cross-reacts with peripheral nervous system (PNS) antigens^20^. The most common subtypes of GBS are acute motor axonal neuropathy (AMAN) and acute inflammatory demyelinating polyneuropathy (AIDP), differentiated by the site of immune-mediated injury^21^. In AMAN, membranes of the nerve axon are the primary targets of cross-reactive anti-ganglioside antibodies while in AIDP the Schwann cell (SC)-produced myelin sheath is the target for damage but the relative roles and specificities of antibody and cellular immune responses are unclear^20^.

Several studies have sought to determine the type of GBS associated with ZIKV infection. Although *in silico* comparison of the ZIKV protein sequence with human proteins has identified shared peptides^22^, there is no evidence of disease-relevant cross reactivity so the pathogenesis of ZIKV-associated GBS remains unclear. The electrophysiological findings from the French Polynesian GBS cases were reported to be compatible with the AMAN subtype of GBS, but the typical AMAN-associated anti-ganglioside antibodies were rarely detected^16^. In Colombia, nerve-conduction studies and electromyography were consistent with the AIDP subtype of GBS^17^ and the electrophysiologic findings from Martinique were also consistent with AIDP further suggesting that SCs were the target for ZIKV-associated damage^18^.

SCs develop from neural crest cells, are the myelinating glial cells of the PNS and play a central role in peripheral nerve function, maintenance and repair. The SC myelin sheath enables saltatory conduction of action potentials by large diameter axons but SCs also ensheath and maintain axons that are not myelinated. In response to nerve injury SCs can trans-differentiate into a proliferating cell capable of secreting inflammatory mediators that enhance macrophage-mediated myelin removal^23^ and can initiate and regulate local immune responses^24,25^. For instance, SCs can be induced to express MHC class I and II molecules, inflammatory cytokines (*e.g*. TNFα, IL-1β, IL-6, LIF, IL-12, IL-18), chemokines (*e.g*. CCL2, CCL3, CXCL10) and nitric oxide synthase (iNOS) in response to damage or toll-like receptor (TLR) stimulation^26–29^. Induction of cytokines, chemokines and cell surface immunoregulatory proteins by infection of SCs could promote development of potentially damaging virus- or host cell-specific immune responses.

Because GBS is associated with ZIKV infection and often occurs when symptoms of ZIKV infection are still present and ZIKV RNA is detectable, it has been postulated that GBS might be directly associated with ZIKV infection of peripheral nerve cells or the immune response to viral antigens expressed by these cells^17,30–32^. To evaluate the potential for direct ZIKV-induced peripheral nerve damage, we assessed the susceptibility of immortalized primary human Schwann cells (hSCs)^33^ to infection with strains of ZIKV from Africa, Asia and Brazil. Infection with ZIKV was compared to infection with flaviviruses YFV 17D and DENV2 that are less commonly associated with GBS^34,35^. All strains of ZIKV and YFV, but not DENV2, replicated well in hSCs and induced innate responses and cytopathic effect.

## Results

### Human Schwann cells are more susceptible to infection with ZIKV and YFV than DENV

**Figure 1:**
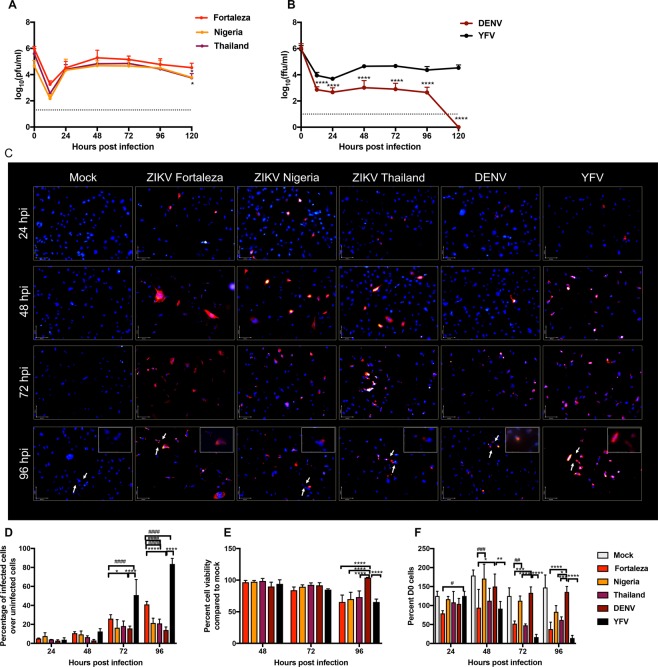
- Replication of ZIKV strains 1968 Nigeria (IBH 30656), 2014 Thailand (SCV0127/14) and 2015 Brazil (Fortaleza), YFV (17D) and DENV2 (NGC) in immortalized human Schwann cells. (**A**) hSC cells were infected with strains of ZIKV from Africa (Nigeria), Asia (Thailand) and Brazil (Fortaleza) (MOI = 5). Virus production was measured by plaque formation in Vero cells. Each value represents the average +/− standard deviation from three independent experiments. *P < 0.05 (Fortaleza vs Nigeria/Thailand). (**B**) hSC cells were infected with DENV2 and YFV (MOI = 5). Virus production was measured by focus formation in Vero cells. Each value represents the average +/− standard deviation from three independent experiments ****P < 0.0001 (DENV vs YFV). (**C**) Immunofluorescence images of mock, ZIKV Fortaleza, ZIKV Nigeria, ZIKV Thailand, DENV, and YFV infected hSCs at 24-96 h after infection (MOI = 5). Cells were stained with pan flavivirus 4G2 antibody followed by anti mouse Alexafluor594 (red). Nuclei were stained with DAPI (blue). The insets show a higher magnification of infected cells (400X). (**D**) The number of infected cells (red) as a percentage of total cells remaining in the culture (blue). Each value represents the average +/− standard deviation from three different fields. *P < 0.05 ****P < 0.0001 DENV vs Fortaleza/YFV, ^####^P < 0.0001 Fortaleza vs Nigeria/Thailand/DENV/YFV (**E**) hSC viability after infection with three strains of ZIKV, YFV and DENV (MOI = 0.1) as determined by MTT assay. Readings taken at 570 nm were plotted as a percentage of the value for mock-infected cells. Each value represents the average +/− standard deviation from four independent infections. ****P < 0.0001 (DENV vs Fortaleza/Nigeria/Thailand/YFV) (**F**) hSC viability after infection with three strains of ZIKV, YFV and DENV (MOI = 5) as determined by trypan blue exclusion. Each value represents the average +/− standard deviation from three independent experiments of the numbers of viable cells compared to d0 expressed as a percentage. *P < 0.05, **P < 0.01, ***P < 0.001, ****P < 0.0001 (DENV vs Fortaleza/Nigeria/Thailand/YFV), ^#^P < 0.05, ^##^P < 0.01, ^###^P < 0.001 (Fortaleza vs Nigeria/YFV).

Immortalized hSCs express SC-characteristic proteins (*e.g*. S100B), transcription factors (*e.g*. Slug, TWIST), cell surface receptors (*e.g*. p75NTR), chemokines (*e.g*. CCL2) and neurotrophic factors (*e.g*. NGF, BDNF, NT-3) and can myelinate axons *in vitro*^36^, but have not been evaluated for susceptibility or response to virus infection. To determine susceptibility of hSCs to flavivirus infection, cells were incubated (MOI = 5) with YFV 17D, DENV2 and three strains of ZIKV: 1968 Nigeria, 2014 Thailand and 2015 Brazil (Fortaleza) chosen to represent the historical shifts of ZIKV as it moved from Africa to Asia and finally the Americas^37^. Supernatant fluids, collected from 0 to 120 h after infection, were analyzed by plaque assay (ZIKV, Fig. 1A) or focus forming assay (YFV and DENV, Fig. 1B). The three ZIKV strains replicated well with the highest virus production by ZIKV Brazil (Fortaleza) that peaked at 48 h and continued through 120 h (Fig. 1A). YFV 17D also replicated in hSCs, but DENV2 infection resulted in little virus production (Fig. 1B).

To further evaluate the effects of ZIKV, YFV and DENV infection of hSCs, cells were stained for expression of viral protein and visualized by immunofluorescence microscopy 24–96 h after infection (Fig. 1C). Percentages of cells remaining in the cultures that were infected were quantified (Fig. 1D) and cell viability was determined by MTT metabolism (Fig. 1E) and trypan blue exclusion (Fig. 1F). The percentages of cells expressing detectable viral protein at 48 h when there is little cytopathic effect were: ZIKV Fortaleza 10.7%; ZIKV Nigeria 9.5%; ZIKV Thailand 6.5%; DENV 3.0%; and YFV 12.5%. At 72 and 96 h the numbers of cells in cultures infected with ZIKV Fortaleza, ZIKV Thailand and YFV were greatly diminished (Fig. 1F) and most of the remaining cells were infected (Fig. 1D). Individual ZIKV and YFV-positive cells showed more extensive expression of viral proteins than DENV2-infected cells (Fig. 1C).

To evaluate the response of hSCs to flavivirus infection, cell viability was determined using the MTT mitochondrial function (Fig. 1E) and trypan blue exclusion cell permeability assays (Fig. 1F). Infection at a low MOI of 0.1 for the MTT assay showed significant cell death at 96 h for all three strains of ZIKV and YFV but not DENV2. Infection at a higher MOI of 5 for the trypan blue assay showed evidence of cell death in ZIKV Fortaleza-infected cells at 24 h ZIKV Thailand-infected cells at 48 h, while many ZIKV Nigeria-infected cells were viable 96 h after infection. Cell death was most extensive for YFV-infected cells and few cells remained by 72 h (Fig. 1D,F) and most of the remaining cells were infected (Fig. 1D). Therefore, hSCs were similarly susceptible to evolutionarily distinct ZIKV strains with the Brazilian strain producing the most virus and the African strain the least cytopathic effect. However, hSCs were comparatively resistant to infection with DENV2.

### ZIKV Fortaleza and YFV infections of human Schwann cells induced expression of innate responses

**Figure 2:**
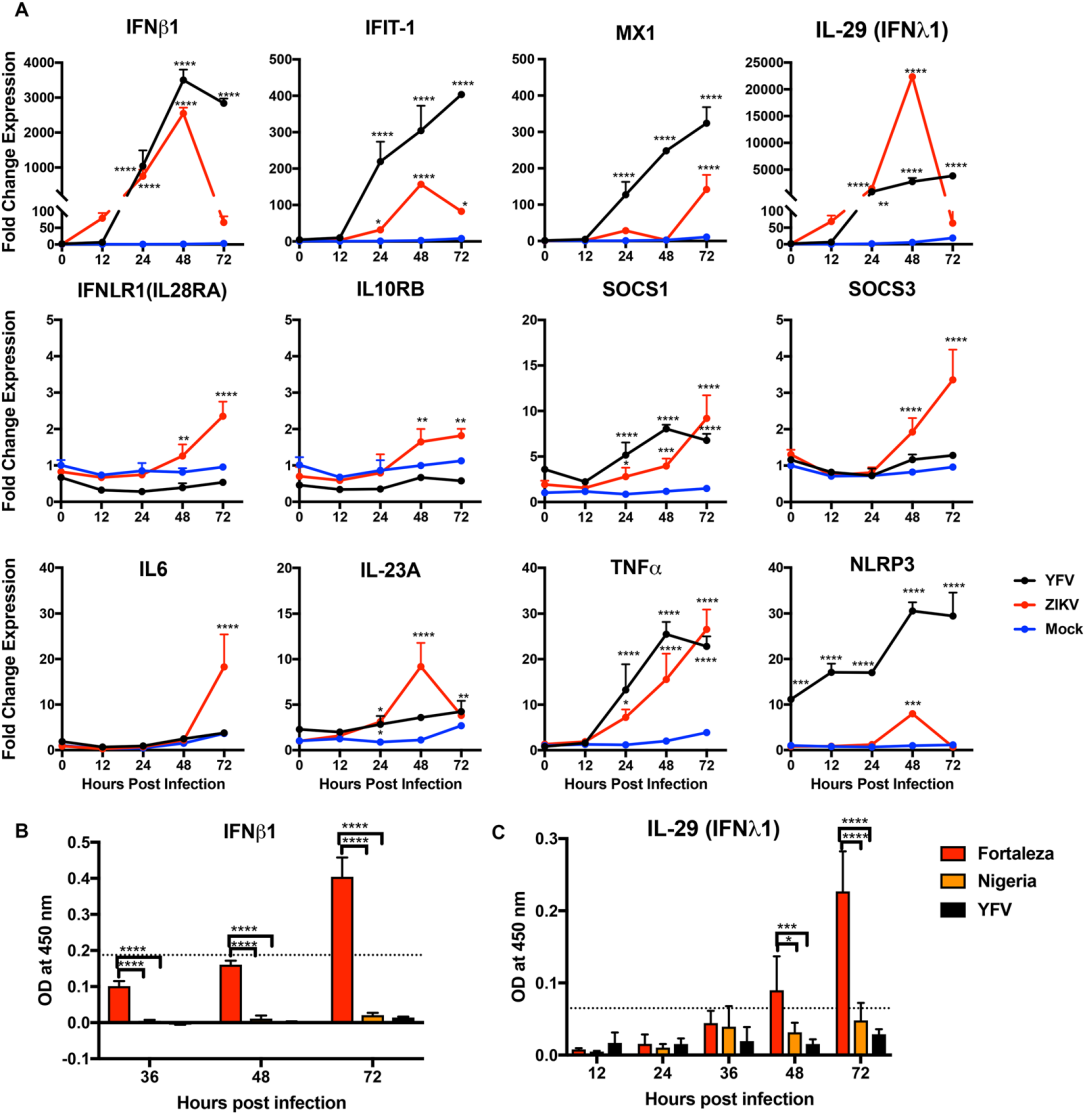
Expression of immune response protein mRNAs and proteins induced by ZIKV and YFV infection of human Schwann cells. (**A**) hSC cells were infected with ZIKV Fortaleza and YFV (17D) (MOI = 5) and mRNA expression was measured by qRT-PCR. ZIKV-infected cells (red) and YFV-infected cells (black) were compared to mock-infected cells (blue). C_T_ values were normalized to *Gapdh* and fold change was calculated relative to uninfected 0-hour (ΔΔC_T_) data. Each value represents the average +/− SD from three independent experiments *P < 0.05, **P < 0.01, ***P < 0.001, ****P < 0.0001 (mock vs infected). (**B,C**) hSC cells were infected with ZIKV (Fortaleza and Nigeria) and YFV (17D) (MOI = 5) and supernatant fluids were collected. Levels of IFNβ1 (**B**) and IFNλ1/IL-29 (**C**) protein were measured by EIA and optical density was plotted. Data are presented as the mean ± SD for three independent infections. *P < 0.05, ***P < 0.001, ****P < 0.0001(Fortaleza vs YFV/Nigeria).

Interferon (IFN) is an important regulator of cell susceptibility to infection. To determine the responses of hSCs to ZIKV Fortaleza and YFV 17D infection, which had similar levels of replication and similar response (Fig. 1), we analyzed expression of mRNAs for IFN and other IFN pathway proteins (Fig. 2). Levels of mRNAs for IFNβ1 and IFN response gene proteins Mx1, a GTPase induced by type I and type III IFNs^38^, and IFN-induced protein with tetratricopeptide repeats (IFIT)-1 were upregulated after infection with both YFV and ZIKV with the greatest response induced by YFV. However, IFN β1 protein production was detectable only in ZIKV Fortaleza-infected cells (119 ± 18.9 pg/ml at 72 h) (Fig. 2B). IFNλ1 (IL-29) was highly induced in ZIKV-infected hSCs at 48 h after infection and in YFV-infected hSCs at 48 h and 72 h (Fig. 2A). ZIKV infection also increased expression of mRNAs for both IFNLR1 and IL10RB components of the IFNλ heterodimeric receptor complex and the SOCS1 and SOCS3 negative regulators of IFN signaling^39^. The levels of IFNλ1 (IL-29) protein at 24 and 36 h were similar, but at 48 and 72 h ZIKV Fortaleza-infected cells produced more IFNλ1 (Fig. 2B). At 48 h protein levels for ZIKV Fortaleza-infected cells were 37.8 ± 10.8 pg/ml and at 72 h were 75.2 ± 16.4 pg/ml for ZIKV Fortaleza and 11.05 pg/ml for ZIKV Nigeria.

To determine whether ZIKV or YFV upregulates hSC expression of other innate response gene mRNAs, levels of mRNAs for representative innate cytokines interleukin (IL)-6, IL-23A and tumor necrosis factor (TNF)-α as well as inflammasome protein NLRP3 were measured (Fig. 2). IL-6 and IL-23A, an innate cytokine related to IL-12 that is associated with induction of autoimmunity^40^, were induced only by ZIKV. Expression of TNFα mRNA was induced in both ZIKV and YFV-infected hSCs. The NLRP3 inflammasome protein mRNA was induced by YFV, but only transiently by ZIKV. Therefore, ZIKV Fortaleza and YFV infections induced overlapping, but distinct innate immune responses in hSCs.

### Expression of potential receptor and immunomodulatory protein mRNAs by hSCs

**Figure 3:**
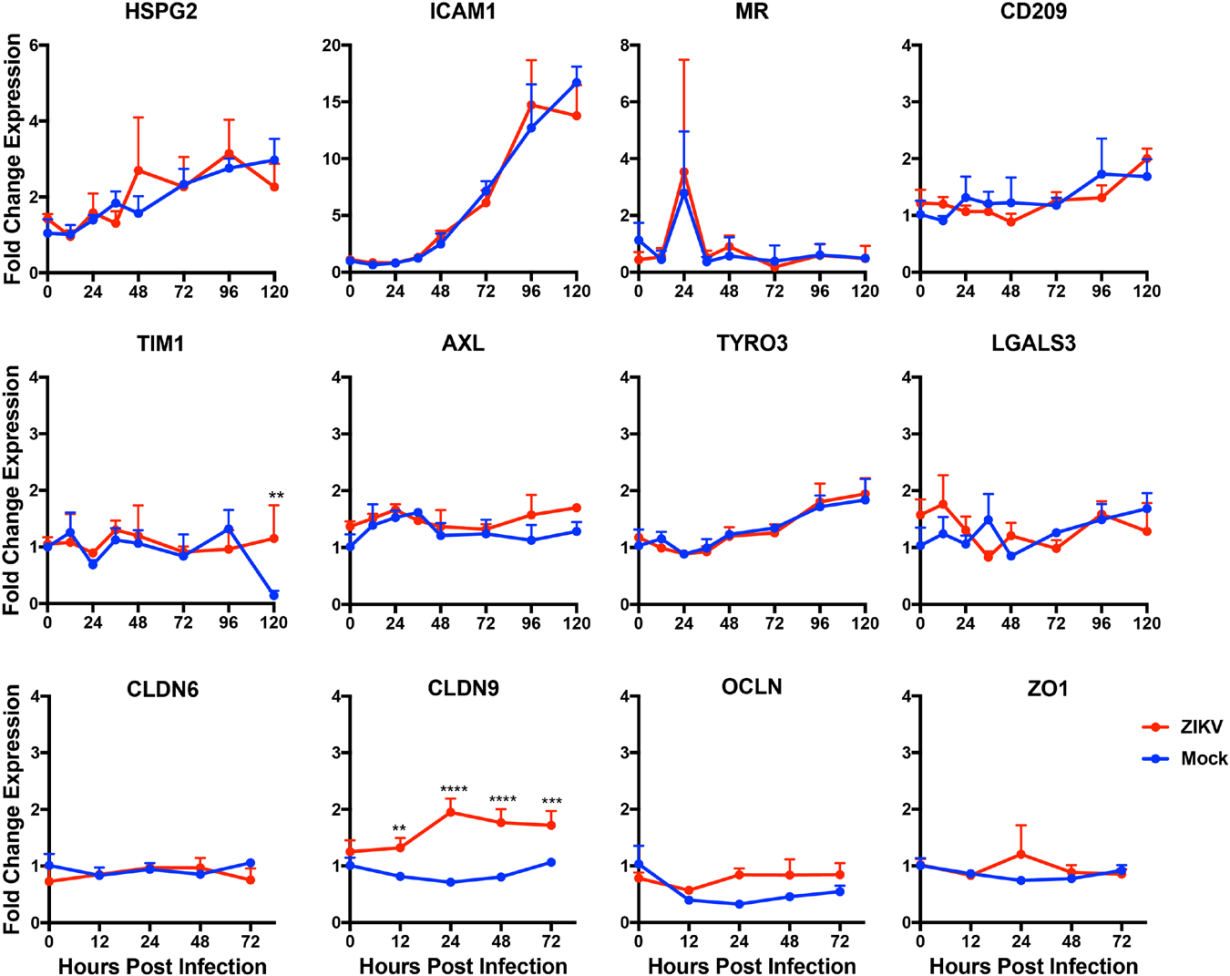
Expression of Fcγ before and after ZIKV infection of human Schwann cells. (**A**) hSC cells were infected with ZIKV Fortaleza at an MOI of 5 and Fcγ receptor mRNA was measured by qRT-PCR. C_T_ values were normalized to *Gapdh*, and fold change was calculated relative to uninfected 0-hour (ΔΔC_T_) data. Each value represents the mean +/− SD from three independent experiments. (**B**) hSC cells were stained with Fcγ receptor-specific antibodies and analyzed by flow cytometry. The blue line in the histogram corresponds to specific staining with the receptor antibody while the red line corresponds to staining with the isotype control antibody. Data are representative of 3 independent sets of experiments.

Like other flaviviruses, ZIKV probably can use multiple receptors for host cell attachment, clathrin-mediated endocytosis, pH-dependent membrane fusion and entry^41–43^. Cell surface molecules of potential importance for ZIKV infection include the C-type lectin receptors CD209 (DC-SIGN), CLEC5a and mannose receptor (MR) and the phosphatidyl serine receptors T-cell immunoglobulin and mucin domain (TIM)-1 and TAM receptors Tyro3 and AXL^42–45^. To begin to identify factors that may facilitate flavivirus infection of hSCs, we analyzed expression of mRNAs for protein receptor families reported to mediate entry (Fig. 3). Baseline transcripts were present for heparan sulfate proteoglycan (HSPG2), an attachment protein for many flaviviruses^42^; intercellular adhesion molecule -1 (ICAM-1); MR, a receptor for DENV^46^ that colocalizes with MHC class II during phagocytosis by SCs^47^; C-type lectin CD209^48,49^; and TIM1 and TAM receptors^42,50^. We also assessed the modulation of receptor expression during culture and infection. HSPG2 and ICAM-1 mRNA expression increased during culture of hSCs, but this was not affected by ZIKV infection. MR mRNA expression showed a transient increase at 24 h in both infected and uninfected cells. There was little change and no effect of infection on expression of CD209, TIM1, AXL or Tyro3. The intercellular cell adhesion molecule (ICAM-1) a cell surface receptor that can be up regulated by IFNγ, IL1β and TNFα in SCs^51^ showed increased mRNA expression during culture but was not modulated by ZIKV infection.

### Expression and modulation of functionally important SC protein mRNAs

Because SC responses to injury can affect peripheral nerve function we also assessed the effect of ZIKV infection on expression of several response-relevant protein mRNAs (Fig. 3). Galectin3 (LGALS3) promotes proinflammatory cytokine expression through the activation of pattern recognition receptor and boosts the phagocytic activity of SCs in injured sciatic nerve in Wallerian degeneration^52^, but levels of mRNA were not modulated by ZIKV infection. Several types of junctional specializations are formed in myelinating SCs between membrane lamellae, act as autotypic junctions and are disrupted during infection of retinal epithelial cells^53,54^. In addition, claudin 1 interacts with the prM protein of DENV to facilitate entry^55,56^. Therefore, we assessed expression of mRNAs for claudins (CLDN) 6 and 9, occludin (OCLN) and zona occludens (ZO)-1. CLDN9 was up regulated in ZIKV-infected hSCs, but other junctional protein mRNAs were not affected by infection.

### Expression of Fcγ receptors (FcγR) by hSCs

**Figure 4:**
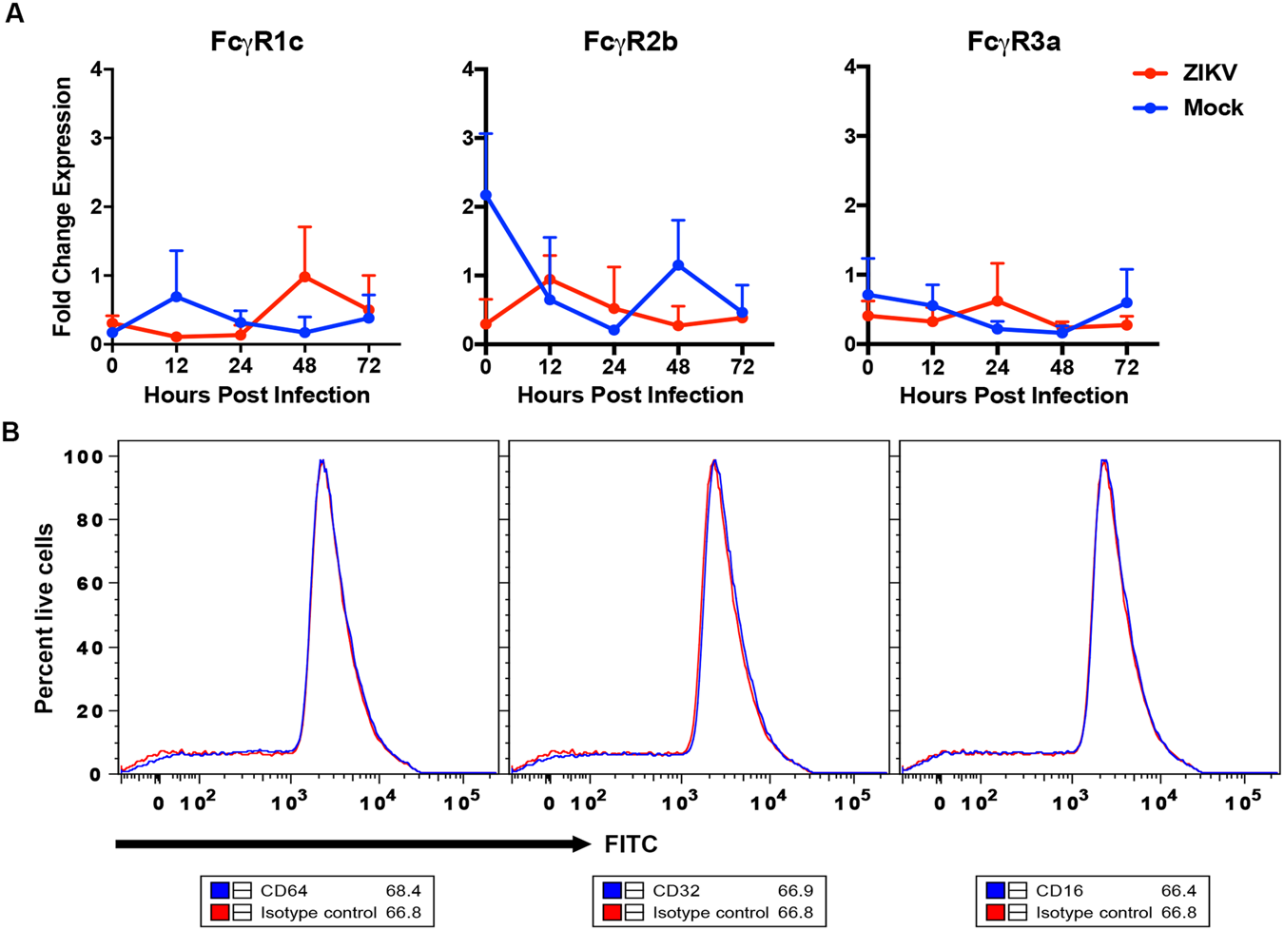
Expression of potential flavivirus receptors. hSC cells were infected with ZIKV (Fortaleza) at an MOI of 5 and levels of cellular receptor mRNA were measured by qRT-PCR. Mock-infected cells (blue) were compared to ZIKV-infected cells (red). C_T_ values were normalized to *Gapdh*, and fold change was calculated relative to uninfected 0-hour (ΔΔC_T_) data. Each value represents the average +/− SD from three independent experiments **P < 0.01, ***P < 0.001, ****P < 0.0001 (mock vs infected).

Cross-reacting non- or sub-neutralizing antibodies can enhance flavivirus infection of FcγR-bearing cells by providing a route of infection for virions coated with non-neutralizing antibodies distinct from binding to the virus cellular receptor and endocytosis^57–61^. SCs in peripheral nerves have been reported to express FcγRII and FcγRIII^62,63^. To determine whether immortalized hSCs express FcγR mRNAs and whether expression is modulated by infection, we assessed the levels of FcγRIc, FcγRIIb or FcγRIIIa mRNAs before and after ZIKV infection (Fig. 4A). Baseline transcripts were detected, but levels were not affected by ZIKV infection. To determine whether FcγR protein was expressed on the cell surface uninfected hSCs were stained with antibodies specific for CD64 (FcγRI), CD32 (FcγRII) and CD16 (FcγRIII) and compared with isotype-specific control antibodies by flow cytometry (Fig. 4B). There was no evidence of FcγR expression on the cell surface.

## Discussion

ZIKV was declared a “Public Health Emergency of International Concern” by the World Health Organization because of the neurological complications of infection: microcephaly and GBS^19^. Evidence that ZIKV triggers the AIDP type of GBS that targets SCs suggested the possibility that ZIKV induces GBS by damaging SCs either directly through infection or through the immune response to viral rather than cellular antigens^17,64^. To assess the potential for ZIKV to infect SCs in comparison with other flaviviruses we analyzed the outcome of ZIKV, YFV and DENV interactions with immortalized primary human SCs^33^. SCs were susceptible to infection with evolutionarily distinct strains of ZIKV and to infection with YFV 17D, but not DENV2. SCs responded to infection with increased expression of mRNAs for IFNβ, IFNλ, IFIT-1, Mx1, NLRP3, IL-23A, IL-6 and TNFα. Baseline transcripts were present for several potential ZIKV receptors and claudin 9 mRNA was increased by infection. hSCs expressed Fcγ receptor mRNAs, but FcγR protein was not detected on the cell surface. Therefore, hSCs are susceptible to ZIKV infection and mount a multifaceted innate response, but are unlikely to be targets for antibody-dependent enhanced infection.

There is substantial variation in susceptibility of human cells to flavivirus infection and ZIKV tropism is generally broader than that of DENV^45,65^. The current studies show that hSCs are susceptible to infection with ZIKV and YFV 17D, but not DENV2. Previous *in vitro* studies have shown ZIKV replication in cells from the skin, lung, placenta, muscle, retina, brain, liver, colon, prostate, testes and kidney^43,53,65–68^. Phylogenetic analysis identifies African and Asian lineages of ZIKV with recent outbreaks due to spread of an Asian strain that had acquired mutations potentially relevant to neurovirulence into the Americas^37,69–71^. However, there is limited evidence of strain-specific variation in ZIKV replication *in vitro* or virulence to explain differences in induction of human neurologic disease^72^. African strains replicate better than Asian strains in 293 T embryonic kidney cells and neural cells and induce more cell death while Asian strains are more likely to establish persistent infection^66,73,74^. American strains replicate better in placental explants and endothelial cells than African strains^45,75^. In our studies Brazilian ZIKV Fortaleza replicated better and induced more rapid cell death in hSCs than African ZIKV Nigeria.

The 17D vaccine strain of YFV also infects a wide range of human cell types *in vitro*^76,77^. Compared to wild type YFV, YFV 17D infects human cells more efficiently, replicates better and induces more robust activation of innate antiviral responses. Studies of YFV 17D infection of HeLa, 293 T, hepatocyte and dendritic cells have shown induction of IFNβ, IL29/IFNλ, IFIT-1, CCL5 and CXCL10 mRNAs that is dependent on a clathrin-independent, dynamin-dependent mode of entry^76^. Our studies showed that YFV 17D also replicated well in hSCs with virus production similar to ZIKV (Fig. 1) and induced strong IFNβ, IFNλ and ISG mRNA synthesis (Fig. 2). However, despite efficient *in vitro* replication in SCs, GBS is a rare complication of YFV 17D immunization potentially because *in vivo* spread of the virus is restricted^35,78^.

Flaviviruses can activate pathogen recognition receptors RIG-I, MDA-5, TLR3 and TLR7 to induce expression of IFN and cytokine genes^43,79,80^. The cellular responses of hSCs to ZIKV Fortaleza and YFV 17D infections showed innate antiviral mRNA responses that were similar. Both viruses induced expression of type I and type III IFNs and IFN-stimulated genes (ISGs) IFIT1 and MX1, as previously described for ZIKV infection of A549 respiratory epithelial cells and glomerular podocytes^67,68^. The IFNβ and ISG response to YFV was greater than to ZIKV, perhaps reflecting the ability of ZIKV to reduce type I IFN induction and NS5 to degrade STAT2 required for IFN signaling^81,82^.

Type III IFN exhibits antiviral and anti-proliferative activities produced through a different receptor but the same downstream signaling pathways as IFNα and IFNβ^83^. Both ZIKV and YFV-infected cells showed significant increases in IL-29 (IFNλ1) mRNA at 48 h although larger amounts of both IFNβ and IFNλ proteins were produced by ZIKV-infected cells than YFV-infected cells perhaps because of better viability (Fig. 1F). Increased expression of the IFNλ heterodimeric receptor complex and negative regulators SOCS1/3 were also observed during ZIKV infection.

The mechanism(s) of ZIKV-induced GBS is not understood. Speculated mechanisms include: Antibody dependent enhancement with DENV sera, an immune mediated mechanism inducing damage through molecular mimicry, cellular mediated inflammation and demyelination induced by complement and macrophage activation, and a direct viral pathogenic effect^64^. Similar mechanisms have been proposed to explain the increased incidence of GBS associated with hepatitis E virus (HEV) infection^84–86^. HEV can replicate in neural cells and virus is often detectable at the onset of GBS, but tropism for SCs has not been examined^87^. Our study of the relationship between ZIKV and GBS, specifically examining hSCs, suggests that GBS could be mediated by pathogen-specific antibodies/T cells or a direct viral effect on SCs rather than cross-reactive antibodies to host proteins/lipids. However, pathologic evaluation of the limited tissue available has not detected ZIKV in peripheral nerves of patients dying with ZIKV-associated GBS^88,89^. The understanding that ZIKV can replicate within hSCs and induces a cellular response that this study demonstrates facilitates a better understanding of the role of ZIKV and GBS in the most recent epidemic.

## Methods

### Cell cultures

Human SCs, a cell line derived from 60–80 day fetal sciatic nerves and immortalized with SV40 large T antigen and human telomerase reverse transcriptase have been previously described and were obtained from Ahmet Hoke (Department of Neurology, Johns Hopkins School of Medicine)^33,36^. hSCs were grown in Dulbecco’s modified Eagle’s medium (DMEM; GIBCO Life Technologies) supplemented with 0.2% glucose, 2mM L-glutamine, 10% heat-inactivated fetal bovine serum (FBS), 2 µM forskolin, penicillin (100 U/ml) and streptomycin (100 µg/ml) at 37 °C in 5% CO_2_. Vero cells (American Type Culture Collection) were grown in DMEM with 10% FBS, 2mM L-glutamine, penicillin and streptomycin at 37 °C in 5% CO_2_. C6/36 *Aedes albopictus* cells (American Type Culture Collection) were grown in DMEM supplemented with 10% FBS and minimal essential amino acids, at 28 °C in 5% CO_2_. Cell viability was determined by trypan blue exclusion.

### Viruses and virus assays

YFV (17D), DENV2 (NGC) and ZIKV strains 2014 Thailand (SCV0127/14) and 2015 Brazil (Fortaleza) were obtained from Anna Durbin (Johns Hopkins Bloomberg School of Public Health). ZIKV strain 1968 Nigeria (IBH 30656) was obtained from Andrew Pekosz (Johns Hopkins Bloomberg School of Public Health). All virus stocks were grown in C6/36 mosquito cells. Stocks and supernatant fluids from infected cells (MOI = 5) were assayed on Vero cells by focus-formation for DENV and YFV and by plaque-formation for ZIKV. Samples serially diluted in DMEM plus 1% FBS were incubated with Vero cell monolayers for an hour. For focus-forming assays, cells were overlaid with 1% methylcellulose in Optimem (Gibco) with 2% FBS, 2 mM glutamine, 50μg/ml gentamicin (Sigma Aldrich) and incubated for 3 days. Cells were fixed with 80% methanol for 10 min, blocked with 5% nonfat milk for 10 min and incubated for 1 h with pan-flavivirus mouse 4G2 monoclonal antibody (purified from hybridoma cells, American Type Culture Collection)^90^ diluted 1:2000 in 5% milk followed by horse radish peroxidase-conjugated goat anti-mouse IgG (KPL) diluted 1:3000 in 5% milk for 1 h. Foci were developed with KPL TrueBlue Peroxidase Substrate (Sera Care). For plaque assays, cells were overlaid with 0.6% Bacto Agar in Modified Eagle Medium (MEM, Gibco), incubated for 5 days, fixed with 10% formaldehyde in PBS and stained with 0.2% crystal violet in 20% ethanol.

### MTT cell viability assay

hSCs were infected with three strains of ZIKV, YFV and DENV (MOI = 0.1). At different times after infection the supernatant fluid was removed and 50μl of MTT reagent (5 mg/ml; thiazolyl blue tetrazolium bromide, Affymetrix USB) in DMEM was added. After 2 h 50μl lysis buffer (20% sodium dodecyl sulfate [Biorad] in 50% N, N′-dimethyl formamide [Applied Biosystems]) was added and incubated for 4 h. Readings at 570 nm were used to calculate the percentage of viable infected cells compared to mock-infected cells.

### Immunofluorescence

hSCs grown on poly-L-lysine-coated cover slips were infected with the three strains of ZIKV, DENV2 and YFV at a MOI of 5. At 24–96 h after infection, cells were fixed with 4% formaldehyde, permeabilized with 0.2% triton X 100 in PBS, and blocked with 5% normal goat serum in PBS. The cells were incubated with pan-flavivirus 4G2 primary antibody (1:1000 in PBS) for 1 h followed by anti-mouse IgG Alexafluor594 (Invitrogen). Nuclei were stained with DAPI. Images were captured using a Zeiss Axio Imager M2 microscope and analyzed using Volocity software. Numbers of infected and uninfected cells in three fields were counted to determine percentages of cells infected for each virus.

### Quantitative real-time RT-PCR (qRT-PCR)

hSCs infected with ZIKV Fortaleza and YFV (MOI = 5) were collected in RLT buffer at 0, 12, 24, 48, and 72 h after infection in triplicate. RNA was isolated from cell lysates using the RNeasy Plus Mini Kit (Qiagen) and quantified using a Nanodrop-1000 spectrophotometer. cDNA was synthesized using the High Capacity cDNA Reverse Transcription Kit (Applied Biosystems) on an Applied Biosystems 2720 thermal cycler with an RNA concentration of 500 ng/µl in a 20 µl total reaction volume. qPCR was performed with the Promega GoTaq qPCR Master Mix for Dye-Based Detection using gene-specific oligonucleotide primers for SYBR Green-based measurements or Applied Biosystems TaqMan probes (Table 1). Applied Biosystems 7500 Real Time PCR System was used under the following conditions: initial hold for 2 min at 50 °C and hold for 10 min at 95 °C followed by 40 cycles to denature for 15 s at 95 °C and anneal for 60 s at 60 °C. All gene expression data were normalized against GAPDH levels and fold change compared to uninfected cells was calculated as 2^−∆∆ct^.

**Table 1:** Primers used for the study.

TaqMan Probes (Applied Biosystems)			
Gene	RefSeq.	Taqman Assay ID	
IFNB1	NM_002176	Hs01077958_s1	
IFIT-1	NM_001270929	Hs01675197_m1	
NLRP3	NM_001079821	Hs00918082_m1	
TNFα	NM_000594	Hs00174128_m1	
IL-29	NM_172140	Hs00601677_g1	
SOCS1	NM_003745	Hs00705164_s1	
SOCS3	NM_003955	Hs02330328_s1	
MX1	NM_001144925	Hs00895608_m1	
**IDT Primers**			
**Gene**	**RefSeq.**	**Forward Primer (5′-3′)**	**Reverse Primer (5′-3′)**
IL-23A	NM_016584	AGCCGCCCGGGTCTT	TCCTTGAGCTGCTGCCTTTAG
IL6	NM_000600	CCAGCTATGAACTCCTTCTC	GCTTGTTCCTCACATCTCTC
IFNLR1	NM_173065	GTAGATGGTTCTGGCACTGAG	GATCTGAAGTATGAGGTGGCA
IL10RB	NM_000628	CTCATCCGACAATGGAAAGGA	AAGTACGCCTTCTCCCCTA
FCGR1C	NR_027484	TGGAGACCTGCAGTAGTGG	CCCAGCTACAGATCACCTC
FCGR2B	NM_004001	GATTAGTGGGATTGGCTGGTT	GTAGTGGCCTTGATCTACTGC
FCGR3A	NM_001127596	TGGGAGATCAGTCCGCAT	TGAGCTAAATCCGCAGGAC
HSPG2	NM_005529	CATAGAGACCGTCACAGCAAG	AGGGCTCGGAAATAAACCATC
ICAM1	NM_000201.2	CAATGTGCTATTCAAACTGCCC	CAGCGTAGGGTAAGGTTCTTG
MR	NM_006039.4	GTCATCATTGTGATCCTCCTG	GATGACCGAGTGTTCATTCTG
CD209	NM_021155.3	TGGACACTGGGGGAGAGTGG	CATGGCCAAGACACCCTGCT
TIM1	NM_001099414	AACAGATGGGAATGACACCG	GAAGCACCAAGACAGAAATACAG
AXL	NM_001699	TTTATGACTATCTGCGCCAGG	TGTGTTCTCCAAATCTTCCCG
TYRO3	NM_006293.3	GCCGCCGCAGGTCTGAAG	GTCAGGCTCCTCCATCCCCT
LGALS3	NM_002306.3	CTGGGAGTATTTGAGGCTCG	TTTCCAGACCCAGATAACGC
GAPDH	NM_002046.5	CCATCACTGCCACCCAGAAGAC	GGCAGGTTTTTCTAGACGGCAG
CLDN1	NM_021101	GATTCTATTGCCATACCATGCTG	TGTATGAAGTGCTTGGAAGACG
CLDN6	NM_021195	GAGACCAGGCCATTCACC	AATTTCCCTTATCTCCTTCGCA
CLDN9	NM_020982	AAAGCGTCCGTAGCATCTG	GTTTCTGTCCTGAGCCTGTT

### Protein quantification by enzyme immunoassay

hSCs were infected with ZIKV Fortaleza, YFV and Nigeria and supernatant fluids were collected at 0, 12, 24, 36, 48 and 72 h after infection. IFN β was measured using the VeriKine Human IFN Beta ELISA kit (PBL Assay Science) and IL-29 (IFNλ1) was measured using the Human IL29 Uncoated ELISA kit (Invitrogen) according to the manufacturer’s instructions. Supernatant fluids from 3 independent infections were tested and data are presented as pg per ml or OD at 450 nm. Assay range was 50 to 4000 pg/ml for IFNβ and 15.6 to 1000 pg/ml for IL-29. The limit of detection is marked as the OD value obtained for lowest concentration of assay range.

### Fcγ receptor staining

hSCs were collected in ice cold PBS and live/dead staining (Invitrogen) was done on ice for 30 min in the dark. Cells were stained with FITC-conjugated mouse antihuman CD64 (FcγRI; BD-560970), CD32 (FcγRII; ebio 11-0329), CD16 (FcγRIII; BD-555406) and mouse IgG1 (isotype control; BD-551954) for 1 h on ice and analyzed on a FACSCanto flow cytometer. Histograms were plotted to determine mean fluorescence intensity.

### Statistics

Data were compared using two-way ANOVA and are presented as mean +/− standard deviation. P < 0.05 was considered significant. All statistical analyses were performed with GraphPad Prism 5.
